# The Examination of Urine for Sugar.—II

**Published:** 1908-02-15

**Authors:** 


### Pathology in General Practice.

### THE EXAMINATION OF URINE FOR SUGAR.—II.

The fallacies attendant on Fehling's reaction depend on two main factors. In the first place, the urine may contain substances other than dextrose capable of reducing cupric oxide; in the second place, there may be present substances which obscure the reaction of dextrose on Fehling's solution, by rendering soluble the product of reduction, cuprous oxide, and preventing its precipitation. The former group includes albumen, maltose, lactose, galactose, uric acid, hippuric acid, urates, and glycuronic acid; these substances, with the exception of glycuronic acid, seldom produce a typical reduction of Fehling's solution, but usually give a greenish-white, or, at times, almost a black precipitate. The latter group includes creatin, mucin, and ammonia.

Of the substances reducing Fehling's solution the most important, from a practical point of view, are albumin, glycuronic acid, uric acid, and urates. Any albumin present must always be removed before applying Fehling's test; this is easily done by heat and filtration. Glycuronic acid, an oxidation product of dextrose, is not infrequently present in normal urine, especially after taking such drugs as morphia, chloral, chloroform, camphor, copaiba, or salicylic acid; it seems to have no pathological significance. Its reaction on Fehling's solution is almost identical with that of dextrose, for which, no doubt, it has been frequently mistaken. Glycuronic acid is not fermented by yeast, and can also be distinguished from dextrose by the phenylhydrazine test. An aqueous solution of uric acid or urates in such quantities as are present in normal urine has a distinct reducing action on Fehling's solution; in urine, however, these bodies must be present in excess before any reduction occurs.

Of the substances inhibiting the reduction of Fehling's solution creatinin seems to be the most important. In health the urine contains 2 to 3 milligrammes of creatinin per c.c., an amount sufficient to counteract the reducing action of 1 per cent. of sugar. Creatin and mucin have a similar, but less powerful, retarding influence. It is probable that the small amount of sugar present in normal urine would be shown by Fehling's reaction but for the combined retarding effect of these three compounds. When the percentage of sugar is slightly increased Fehling's solution often assumes a dirty-green colour, or there may be a greenish precipitate. Another important interfering agent is ammonia. This must be removed by boiling or neutralisation, when the urine is highly ammoniacal.

2. *The Fermentation Test.*—This depends on the conversion of any sugar present into alcohol and $CO_2$ by the yeast-ferment. An inverted test-tube (or special fermentation-tube) is filled with urine, and to this is added a small piece of yeast of the size of a pea. The urine is then incubated at a temperature of 25° to 30° C. for twelve hours, when any gas evolved will have collected at the top of the tube. For two reasons this test is unreliable when dealing with small quantities of sugar—*e.g.* less than 1 per cent. First, the $CO_2$ given off may become dissolved in the urine, in which it is soluble to a considerable extent. Secondly, the "pressed" yeast of commerce often contains some starch, as may be seen by testing with iodine. It is always advisable, therefore, to perform a control experiment with yeast and water, to make sure that no gas is formed under these conditions.

3. *The Phenylhydrazine Test* depends on the formation of crystals of phenyl dextrosazone by the interaction of dextrose and phenylhydrazine. To 50 c.c. of the urine add 2 grammes of sodium acetate and 1 gramme of phenylhydrazine hydrochloride, and heat the mixture in a water-bath to 100° C. for thirty minutes; stand aside till cold, and examine any sediment under a microscope. If the urine contains sugar there will be seen clusters of radiating, needle-shaped crystals of a bright yellow colour. Glycuronic acid also forms a crystalline osazone, but the melting-point of these crystals is 125° C., compared with 204° C. for dextrosazone. Lactose and maltose form similar compounds, with melting-points of 200° C. and 190° C. respectively. The test cannot be considered complete unless the melting-point of the crystals is determined.

4. *The Safranin Test.*—Safranin is a red powder dissolving in water to give a deep red solution. On boiling this with glucose, in alkaline solution, reduction occurs and the red colour disappears, leaving a clear yellow solution. For urinary work a solution of 1 gramme of safranin to the litre of water is the best; a normal solution of caustic soda (40 grammes to the litre) is also required. With these reagents it may be reckoned that each 2 c.c. of safranin reduced—*i.e.* decolorised—by 2 c.c. of urine represents approximately .1 per cent. of sugar. Inasmuch as healthy urine contains .1 or even .2 per cent. of sugar, it follows that 2 to 4 c.c. of safranin may be decolorised under normal conditions; any further reduction must be regarded as pathological. The exact technique is as follows: Mix in a test-tube 2 c.c. each of safranin solution, alkaline solution, and urine, and boil. When the colour has been discharged, as is almost certain to happen, add a further 2 c.c. of safranin and again boil; once more reduction may occur without any pathological import. Continue adding safranin until the red colour no longer disappears. The solution should not be shaken after boiling, as oxidation by the air readily occurs. Precipitation of a leuco-derivative may cause a white cloud, but this does not affect the reaction.

The advantages of the safranin test are (i) that it is not affected by creatinin, creatin, mucin, urates, or albumin; (ii) that it never gives a doubtful result, the change of colour being definite and complete; (iii) that it is not only a qualitative test, but gives a fairly accurate idea of the quantity of sugar present. On the other hand, it fails to distinguish between glycuronic acid and dextrose; this can only be done by the fermentation or phenylhydrazine tests.

